# Development and Validation of a Score for Screening Suicide of Patients With Neuroendocrine Neoplasms

**DOI:** 10.3389/fpsyt.2021.638152

**Published:** 2021-06-11

**Authors:** Lili Lu, Yuru Shang, Dietmar Zechner, Christina Susanne Mullins, Michael Linnebacher, Xianbin Zhang, Peng Gong

**Affiliations:** ^1^Department of General Surgery, Molecular Oncology, and Immunotherapy, Rostock University Medical Center, Rostock, Germany; ^2^Department of Plastic Surgery, Southern University of Science and Technology Hospital, Shenzhen, China; ^3^Institute for Experimental Surgery, Rostock University Medical Center, Rostock, Germany; ^4^Department of General Surgery, Carson International Cancer Research Center, Shenzhen University General Hospital, Shenzhen University Clinical Medical Academy, Shenzhen, China; ^5^Guangdong Key Laboratory for Biomedical Measurements and Ultrasound Imaging, School of Biomedical Engineering, Shenzhen University Health Science Center, Shenzhen, China; ^6^Guangdong Key Laboratory of Regional Immunity and Diseases, Shenzhen University Health Science Center, Shenzhen, China

**Keywords:** neuroendocrine neoplasms, suicide, score, proportionate mortality ratio, risk factors

## Abstract

**Background:** If the diagnosis of neuroendocrine neoplasm (NEN) increases the risk of patients to commit suicide has not been investigated so far. Identifying NEN patients at risk to commit suicide is important to increase their life quality and life expectancy.

**Methods and findings:** Cancer cases were extracted from the Surveillance, Epidemiology, and End Results program and were divided into the NEN and the non-NEN cohorts. Subsequently, the NEN patients were randomly split into a training data set and a validation data set. Analyzing the training data set, we developed a score for assessing the risk to commit suicide for patients with NEN. In addition, we validated the score using the validation data set and evaluated, if this score could also be applied to other cancer entities by using the test data set, a non-NEN cohort. The odds ratio (OR) of suicide between NEN and non-NEN patients was determined. Moreover, the performance of a score was evaluated by the receiver operating characteristic curve and the area under the curve (AUC). Compared to non-NEN, NEN significantly increased the risk of suicide to 1.8-fold (NEN vs. non-NEN; OR, 1.832; *P* < 0.001). In addition, we observed that age, gender, race, marital status, tumor stage, histologic grade, surgery, and chemotherapy were associated with suicide among NEN patients; and a synthesized score based on these factors could significantly distinguish suicide individuals from non-suicide individuals in the training data set (AUC, 0.829; *P* < 0.001) and in the validation data set (AUC, 0.735; *P* < 0.001). This score also had a good performance when it was assessed by the test data set (AUC, 0.690; *P* < 0.001). This demonstrates that the score might also be applicable to other cancer entities.

**Conclusions:** This population-based study suggests that NEN patients have a higher risk of suicide than non-NEN patients. In addition, this study provided a score, which can identify NEN patients at high-risk of committing suicide. Thus, this score in combination with current screening and prevention strategies for suicide may improve life quality and life expectancy of NEN patients.

## Introduction

Neuroendocrine neoplasm (NEN) arises from neuroendocrine cells and is commonly observed in lung, stomach, intestines, and pancreas (1, 2). Recent evidence proved that the incidence of lung and gastroenteropancreatic NEN increased 6.4-fold from 1973 to 2012 in the USA (3). Interestingly, distinct from other tumors, NEN is indolent. In addition, the survival time of these patients is longer than that of patients suffering from adenocarcinoma or squamous cell carcinoma (3). Hence, improving the surveillance and quality of life after diagnosis of NEN has become an important clinical issue.

Suicide has developed into an enormous public health problem all around the globe, and it is one of the leading causes of death in the USA (4–6). Notably, several studies suggest that the suicide incidence of cancer survivors is significantly higher than that of patients suffering from chronic diseases or in the general population (7, 8). However, suicide can potentially be prevented, when individuals at risk for suicide are identified and proper psychological support is given. A mandatory prerequisite is the effective recognition of patients at elevated risk of suicide.

Previous studies suggest that NEN is positively associated with psychiatric disorder symptoms, such as depression, anxiety, or psychosis (9–11). In addition, it is well-known that psychiatric disease is a master contributor to suicides (12). Thus, we hypothesized that NEN may increase the risk of suicide when directly compared to non-NEN. However, to our knowledge, no study addressing this issue has been published. Even though several studies have emphasized the screening of anxiety and depression (13), and some studies have investigated the risk factors of suicide (14–16), none of them provided and evaluated a strategy, which can effectively identify NEN patients at an elevated risk of suicide.

Thus, the primary purpose of this study was to evaluate if NEN increases the risk of suicide. Secondly, we developed and evaluated a score, which may identify NEN patients at high risk for committing suicide. In this study, we observed that NEN significantly increase the risk of suicide, and we developed and validated a score to identify patients at high-risk for committing of suicide. The score in combination with current screening and psychological management of potential suicides might help to prevent or at least reduce suicidal death in patients suffering from neuroendocrine neoplasms.

## Patients and Methods

### Data Source and Study Population

Surveillance, Epidemiology, and End Results (SEER) program is an authoritative source of cancer patients' information in the USA. Dependent on the number of registries, the SEER program has four databases: SEER 9, SEER 13, SEER 18, and SEER 21. This study was performed with the data obtained from SEER 18, which includes ~28% of the USA population (17).

**Figure 1:**
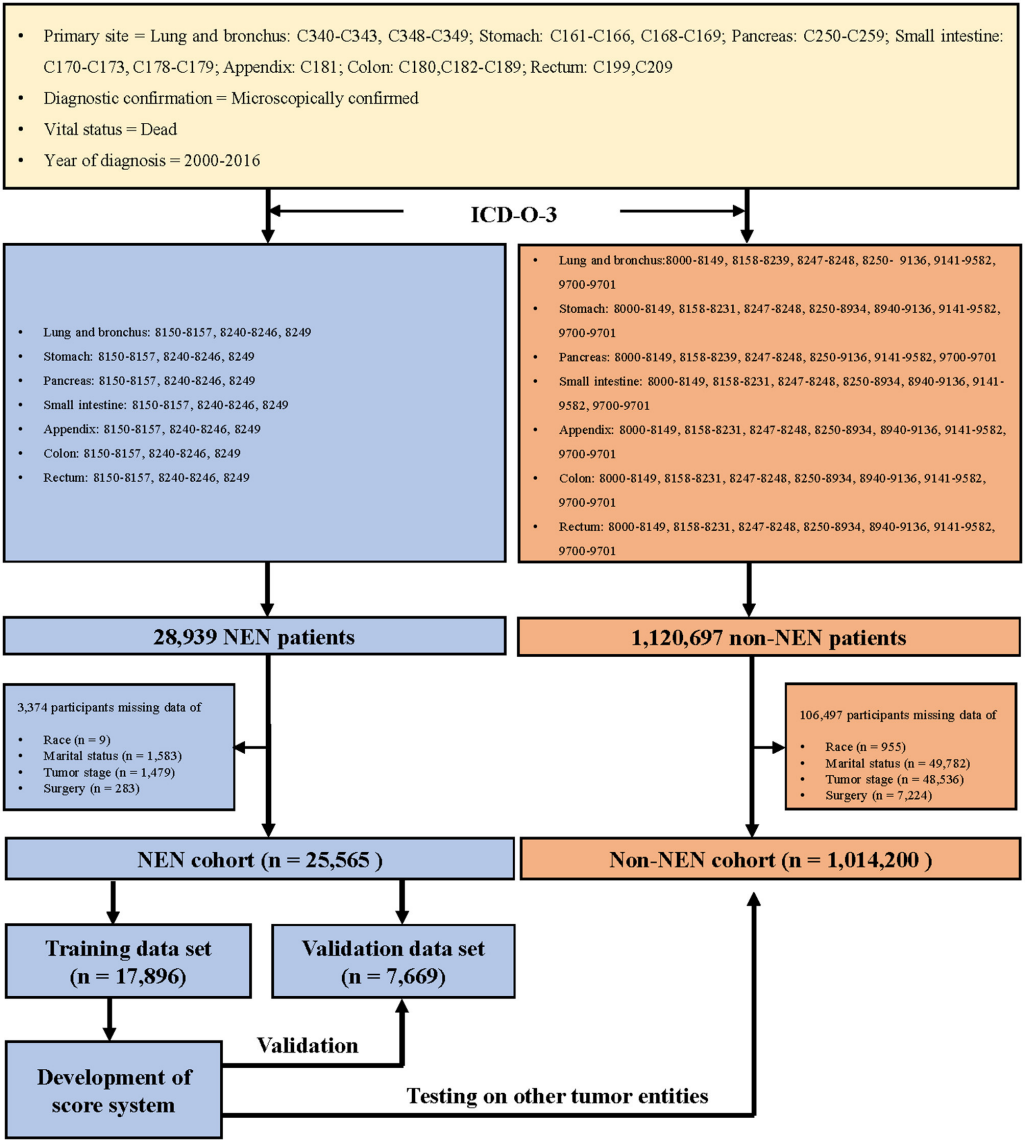
Flow chart of patient inclusion.

We collected the information of patients who were diagnosed with cancer during the years 2000 to 2016, with the support of SEER^*^Stat 8.3.6 version (18), and the topography codes (3) (lung and bronchus: C340–C343, C348–C349; stomach: C161–C166, C168–C169; pancreas: C250–C259; small intestine: C170–C173, C178–C179; appendix: C181; colon: C180 and C182–C189; rectum: C199 and C209). In addition, the patients were classified as NEN or non-NEN patients with the help of the International Classification of Disease for Oncology codes, 3rd edition (3) (Figure 1).

### Study Variables

Notably, the SEER program does not record the suicidal ideation or previous suicide attempts. We defined suicidal behavior dependent on the code of 50,220, which suggested that the patients died of suicide and self-inflicted injury (16). In order to determine the proportionate mortality ratio (PMR) of suicide, we identified the dead cases from the SEER program.

In order to determine the risk factors of suicide, we defined the following variables as covariates: population characteristics (age at diagnosis, gender, race, and marital status); tumor characteristics (tumor stage and histologic grade), and treatment (surgery and chemotherapy). Notably, the histologic grade of tumors was not defined according to the recommendations of WHO and the European Neuroendocrine Tumor Society classification (19, 20). It was determined based on the morphological description in the pathology reports. For example, SEER grade I suggested that tumors were classified as well-differentiated; grade II suggested that tumors were classified as moderately differentiated; grade III suggested that tumors were classified as poorly differentiated, and grade IV suggested that tumors were classified as undifferentiated or anaplastic (17).

### Statistical Analysis

**Figure 2:**
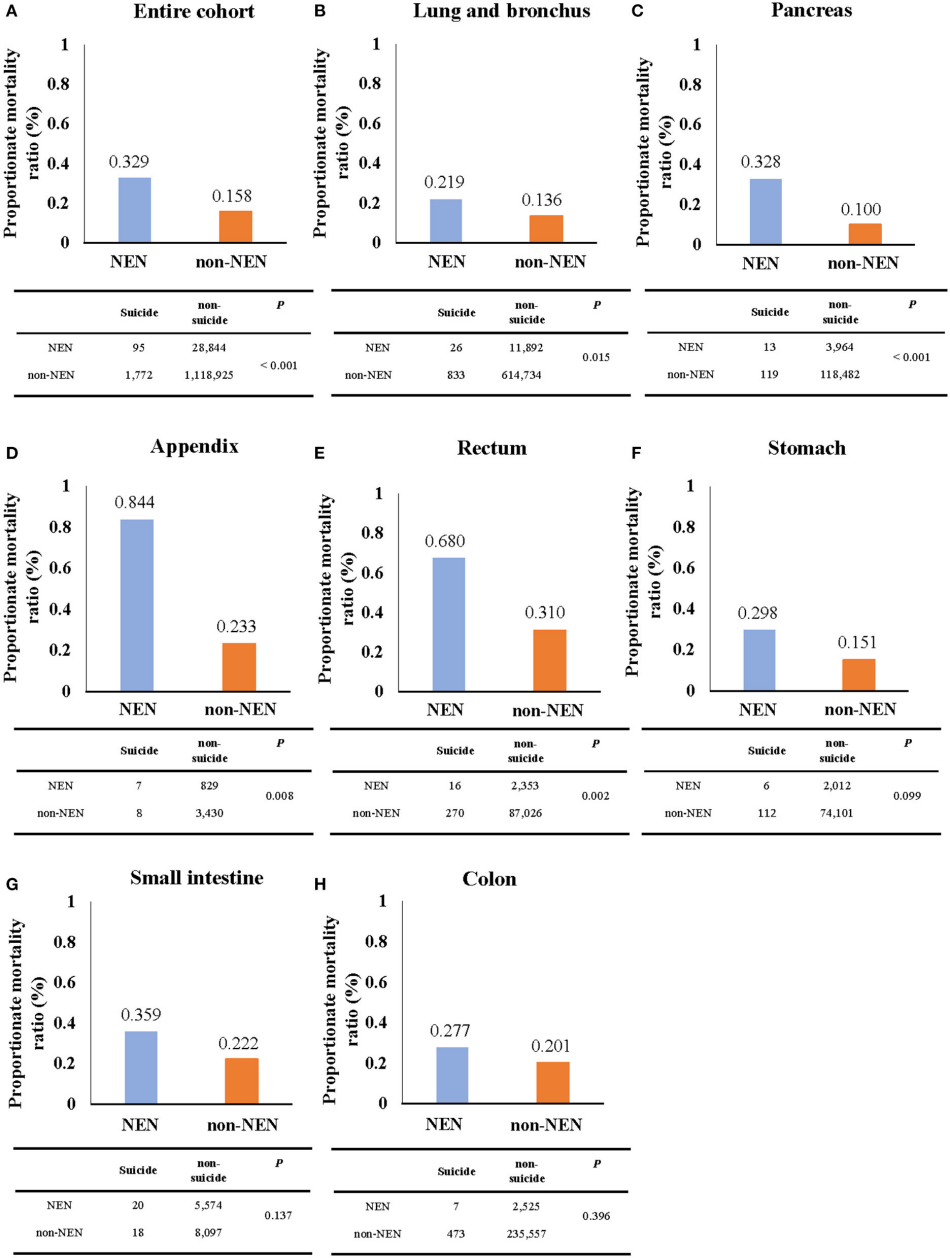
The proportionate mortality ratio (PMR) of suicide. The PMR of suicide of NEN patients was significantly higher than that of non-NEN patients in cohorts with all listed types of cancer **(A)** as well as only lung and bronchus **(B)**, pancreatic **(C)**, appendix **(D)**, and rectum cancer **(E)**. However, no significant difference for PMR of suicide between NEN and non-NEN patients was found, when analyzing stomach cancer **(F)**, small intestine cancer **(G)**, and colon cancer cohorts **(H)**. The *P*-values were determined by Chi-squared test, and *P* < 0.05 indicates a significant difference.

The mortality was calculated using SEER^*^Stat software and presented as per 100,000 population (Supplementary Table 1). The PMR of suicide was calculated by the following formula: (the number of patients committing suicide/the number of patients died) × 100% (Figure 2). The difference of the ratio between the NEN cohort and the non-NEN cohort was evaluated by Chi-squared test. In order to evaluate if NEN was a risk factor for committing suicide, a univariate logistic regression was performed, and the variables which have *P* < 0.05 were included in the multiple logistic regression (enter method, Table 1).

**Table 1:** Univariate and multivariate logistic regression of suicide in entire cohort.

**Variables**		***n***	**Univariate logistic regression**			**Multivariate logistic regression**		
			**OR**	**95% CI**	***P-*value**	**OR**	**95% CI**	***P-*value**
Tumor type	Non-NEN	1,120,697		Ref			Ref	
	NEN	28,939	2.080	1.691–2.557	<0.001	1.832	1.460–2.300	<0.001
Age (years)			0.987	0.984–0.991	<0.001	0.983	0.979–0.987	<0.001
Gender	Female	526,142		Ref			Ref	
	Male	623,494	5.733	5.006–6.565	<0.001	5.735	4.989–6.592	<0.001
Race	Black	137,029		Ref			Ref	
	White	936,122	4.289	3.301–5.572	<0.001	4.816	3.701–6.267	<0.001
	API	69,355	3.376	2.441–4.669	<0.001	3.966	2.862–5.495	<0.001
	AIAN	6,146	3.463	1.715–6.993	0.001	3.551	1.757–7.176	<0.001
	Unknown	984	9.639	3.493–26.602	<0.001	9.289	3.354–25.726	<0.001
Marital status	Married	583,062		Ref			Ref	
	Single	114,360	1.194	1.047–1.361	0.008	1.387	1.211–1.588	<0.001
	Widowed	237,876	0.522	0.450–0.606	<0.001	1.003	0.857–1.174	0.969
	Unmarried	132,789	1.258	1.102–1.436	0.001	1.602	1.400–1.833	<0.001
	Unknown	51,549	1.137	0.925–1.398	0.223	1.267	1.027–1.561	0.027
Primary sites	Colon	238,311		Ref			Ref	
	Lung	627,866	0.681	0.609–0.762	<0.001	0.859	0.609–0.762	0.025
	Stomach	53,081	0.534	0.406–0.703	<0.001	0.611	0.406–0.703	0.001
	Pancreas	122,722	0.535	0.441–0.648	<0.001	0.716	0.441–0.648	0.002
	Small intestine	13,717	1.379	0.991–1.920	0.057	0.986	0.991–1.920	0.937
	Appendix	4,274	1.749	1.045–2.926	0.003	1.396	1.045–2.926	0.213
	Rectum	89,665	1.589	1.372–1.840	<0.001	1.406	1.207–1.638	<0.001
Tumor stage	Distant	580,098		Ref			Ref	
	Regional	308,184	1.779	1.587–1.994	<0.001	1.620	1.426–1.840	<0.001
	Localized	204,954	2.710	2.419–3.036	<0.001	2.145	1.873–2.456	<0.001
	Unknown	56,400	1.654	1.336–2.047	<0.001	1.597	1.283–1.989	<0.001
Grade	IV	45,350		Ref		-		
	III	278,233	1.511	1.111–2.056	0.009			
	II	303,868	2.162	1.598–2.924	<0.001			
	I	54,067	2.483	1.770–3.482	<0.001			
	Unknown	468,117	1.337	0.988–1.810	0.060			
Surgery	No	685,938		Ref			Ref	
	Yes	422,799	2.037	1.855–2.237	<0.001	1.256	1.104–1.428	0.001
	Unknown	40,899	1.984	1.600–2.460	<0.001	1.612	1.296–2.004	<0.001
Chemotherapy	Yes	474,942		Ref			Ref	
	No/unknown	674,694	1.466	1.330–1.615	<0.001	1.324	1.182–1.484	<0.001

*API, Asian/Pacific Islander; AIAN, American Indian/Alaska Native; Ref, reference; OR, odds ratio; 95% CI, 95% confidence interval*.

**Figure 3:**
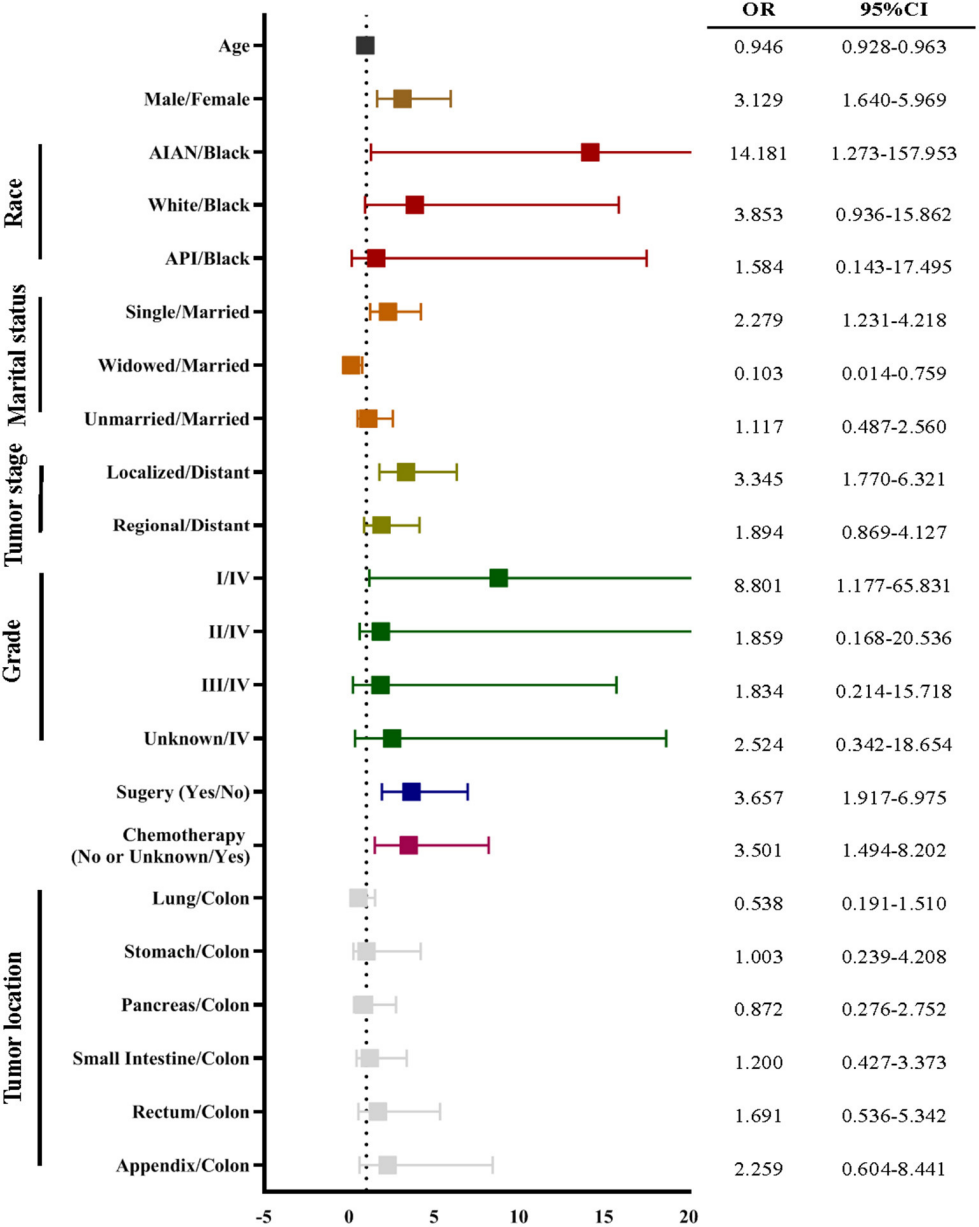
The univariate logistic regression of suicide using the training data set. The univariate logistic regression demonstrated that age, gender, race, marital status, cancer stage, cancer grade, surgery, and chemotherapy were associated with suicide of neuroendocrine neoplasm (NEN). The odds ratio (OR) was presented by a square, and the 95% confidence interval (CI) was presented by the horizontal lines with bars.

To develop the score, we followed the suggestion of the TRIPOD statement (21), 70 and 30% of the NEN patients were randomly split into a training data set and a validation data set, respectively (for basic characteristics of patients in both data sets see Table 2). To determine the risk factors for committing suicide, univariate logistic regression analyses were performed using the training data set; the odds ratios (ORs) and 95% confidence intervals (CIs) of variables were calculated (Figure 3). The β coefficient (β_*i*_) of each category was determined by multiple logistic regression analysis (Table 3). In order to determine the weight of each category, the age was converted into a categorical variable, and the weight was assigned as the value of average age (22, 23). In addition, for the other categorical variables such as gender, race, marital status, tumor stage, histologic grade, surgery, and chemotherapy, the weight of the reference category (W_*iRef*_) was defined as 0 and the weight of the other categories (W_*ij*_) were assigned as 1. Subsequently, the weight of each category was adjusted by the following formula: β_*i*_ × (W_*ij*_–W_*iRef*_). In order to determine the constant score B (Table 3) of the system, we defined B to reflect the decrease of risk when age has increased 10 years (22): B = −0.05 × 10 = −0.5. Finally, the score of each category was computed by β_*i*_ × (W_*ij*_–W_*iRef*_)/B (22–24).

**Table 2:** Basic characteristics of patients in the training data set and the validation data set.

**Variables**		**Training cohort**		**NENs Validation**		***P*-value**
		***n* = 17,896**	**%**	***n* = 7,669**	**%**	
Age (years)		67.56 ± 12.56		67.54 ± 12.39		0.599^&^
Gender	Male	9,244	51.7	3,986	52.0	0.637^#^
	Female	8,652	48.3	3,683	48.0	
Race	White	14,712	82.2	6,256	81.6	0.239^#^
	Black	2,356	13.2	1,015	13.2	
	API	744	4.2	362	4.7	
	AIAN	84	0.5	36	0.5	
Marital status	Married	9,829	54.9	4,241	55.3	0.872^#^
	Single	2,474	13.8	1,068	13.9	
	Widowed	3,392	19.0	1,421	18.5	
	Unmarried	2,201	12.3	939	12.2	
Primary sites	Lung and bronchus	7,591	42.4	3,304	43.1	0.347^#^
	Stomach	940	5.3	392	5.1	
	Pancreas	2,523	14.1	1,087	14.2	
	Small intestine	3,407	19.0	1,399	18.2	
	Appendix	559	3.1	219	2.9	
	Colon	1,572	8.8	662	8.6	
	Rectum	1,304	7.3	606	7.9	
Tumor stage	Localized	4,879	27.3	2,100	27.4	0.923^#^
	Regional	3,637	20.3	1,570	20.5	
	Distant	9,380	52.4	3,999	52.1	
Grade	I	2,303	12.9	978	12.8	0.126^*^
	II	1,140	6.4	499	6.5	
	III	2,889	16.1	1,279	16.7	
	IV	1,059	5.9	478	6.2	
	Unknown	10,505	58.7	4,435	57.8	
Surgery	Yes	8,549	47.8	3,658	47.7	0.916^#^
	No	9,347	52.2	4,011	52.3	
Chemotherapy	Yes	5,600	31.3	2,325	30.3	0.122^#^
	No/unknown	12,296	68.7	5,344	69.7	

*API, Asian/Pacific Islander; AIAN, American Indian/Alaska Native*.

& *The continuous variable (age) was presented as mean ± SD and P-value was determined by student t-test*.

* *The ordinal variable (grade) was presented as frequencies and proportions, and P-value was determined by Mann-Whitney U-test*.

# *The ordinal variables (gender, race, marital status, primary site, stage, surgery, and chemotherapy) were presented as frequencies and proportions, and P-value was determined by Chi-Square test*.

**Table 3:** Risk score of NEN cases in the training data set.

**Risk factors**	**Categories**	**β*_***i***_***	**Weight (W*_***ij***_*)**	**Adjust weight β*_***i***_* (W*_***ij***_*–W*_***iRef***_*)**	**Risk score β*_***i***_* (W*_***ij***_*–W*_***iRef***_*)/B**
Age (years)	60–69 (Ref)		64.5 (W*_1*Ref*_*)	0	0
	9–19	–0.05	14	2.52	5
	20–29		24.5	2.16	4
	30–39		34.5	1.62	3
	40–49		44.5	1.08	2
	50–59		54.5	0.54	1
	70–79		74.5	–0.54	–1
	80–89		84.5	–1.08	–2
	90–99		94.5	–1.62	–3
Gender	Female (Ref)		0 (W_2Ref_)	0	0
	Male	1.03	1	1.03	2
Race	Black (Ref)		0 (W*_3*Ref*_*)	0	0
	White	1.88	1	1.88	3
	API	0.93	1	0.93	2
	AIAN	2.97	1	2.97	5
Marital status	Married (Ref)		0 (W*_4*Ref*_*)	0	0
	Single	0.59	1	0.59	1
	Widowed	–1.33	1	–1.33	–2
	Unmarried	0.22	1	0.22	0
Tumor stage	Distant (Ref)		0 (W*_5*Ref*_*)	0	0
	Regional	0.14	1	0.14	0
	Localized	0.62	1	0.62	1
Grade	IV (Ref)		0 (W*_6*Ref*_*)	0	0
	III	0.58	1	0.58	1
	II	0.02	1	0.02	0
	I	1.37	1	1.37	3
	Unknown	0.50	1	0.50	1
Surgery	No (Ref)		0 (W*_7*Ref*_*)	0	0
	Yes	0.60	1	0.60	1
Chemotherapy	Yes (Ref)		0 (W*_8*Ref*_*)	0	0
	No/Unknown	1.03	1	1.03	2

*API, Asian/Pacific Islander; AIAN, American Indian/Alaska Native; Ref, reference*.

In order to evaluate and validate this score, the performance of this strategy was determined by receiver operating characteristic (ROC) curve analysis using the training data set, the validation data set, and non-NEN cohort, respectively. The area under the curve (AUC) was presented. When the AUC is higher than 0.5, it suggests that the score can discriminate between suicidal patients and non-suicidal patients. All statistical analyses were performed using SPSS 19.0 (IBM, Armonk, New York), and *P* < 0.05 was considered to indicate statistical significance.

## Results

### NEN Increases the Risk of Suicide

During the period of 2000–2016, 95 NEN patients (mortality, 0.00629/100,000; 95% CI, 0.00503/100,000-0.00776/100,000) and 1,773 non-NEN patients (mortality, 0.13089/100,000; 95% CI, 0.12483/100,000-0.13718/100,000) died of suicide (Figure 2A and Supplementary Table 1). Interestingly, we observed that the PMR of the NEN cohort was over 2-fold higher than that of the non-NEN cohort (Figure 2A). In addition, we evaluated the PMR in different tumor sites. Again, we observed that the PMR of NEN patients was higher than that of non-NEN patients in lung and bronchus cancer (Figure 2B), pancreatic cancer (Figure 2C), cancer of the appendix (Figure 2D), rectum cancer (Figure 2E), stomach cancer (Figure 2F), small intestine cancer (Figure 2G), and colon cancer (Figure 2H). In order to evaluate if NEN is a risk factor for suicide, the multiple logistic regression analysis was subsequently performed. We observed that NEN significantly increased the risk of suicide by 1.8-fold in comparison to non-NEN (Table 1).

### Development of a Score for Predicting Suicides Among NEN Patients

In order to develop a score for predicting suicide, we excluded 3,374 participants, because the data of race, marital status, tumor stage, or surgical treatment were not recorded in the SEER program. Thus, 25,565 NEN patients were included in the subsequent analyses, and were randomly split into a training data set and a validation data set. The clinicopathological characteristics of each data set were summarized in Table 2. We did not observe significant differences of any clinicopathological characteristics between the two data sets. The ORs and 95% CIs of each variable were calculated using the training data set. We observed that being young, male, American Indian/Alaska Native, single, localized tumor, well-differentiated, surgery, and non-chemotherapy were significantly (*P* < 0.05) associated with suicide (Figure 3). In order to develop the score system, the β_*i*_ and the weight of each category were determined as indicated in Table 3, and the score of each category was determined by β_*i*_ × (W_*ij*_-W_*iRef*_)/B (Tables 3, 4).

**Table 4:** A SEER scale for screening suicide of NEN patients.

**Population characteristics**		**Points**
Age (years)	□ 9–19	5
	□ 20–29	4
	□ 30–39	3
	□ 40–49	2
	□ 50–59	1
	□ 60–69	0
	□ 70–79	–1
	□ 80–89	–2
	□ 90–99	–3
Gender	□ Male	2
	□ Female	0
Race	□ White	3
	□ Black	0
	□ Asian/Pacific Islander	2
	□ American Indian/Alaska Native	5
Marital status	□ Married	0
	□ Single	1
	□ Widowed	–2
	□ Unmarried	0
**Tumor characteristics**		
Tumor stage	□ Localized	1
	□ Regional	0
	□ Distant	0
Grade	□ I; well-differentiated	3
	□ II; moderately differentiated	0
	□ III; poorly differentiated	1
	□ IV; undifferentiated or anaplastic	0
	□ Unknown	1
**Treatments**		
Surgery	□ Yes	1
	□ No	0
Chemotherapy	□ Yes	0
	□ No/Unknown	2
**Total score**		
□ **Low risk (Total score:** **−5–9 points)**		
□ **High risk (Total score: 10–20 points)**		

### Validation of the Score for Predicting Suicide Among NEN

**Figure 4:**
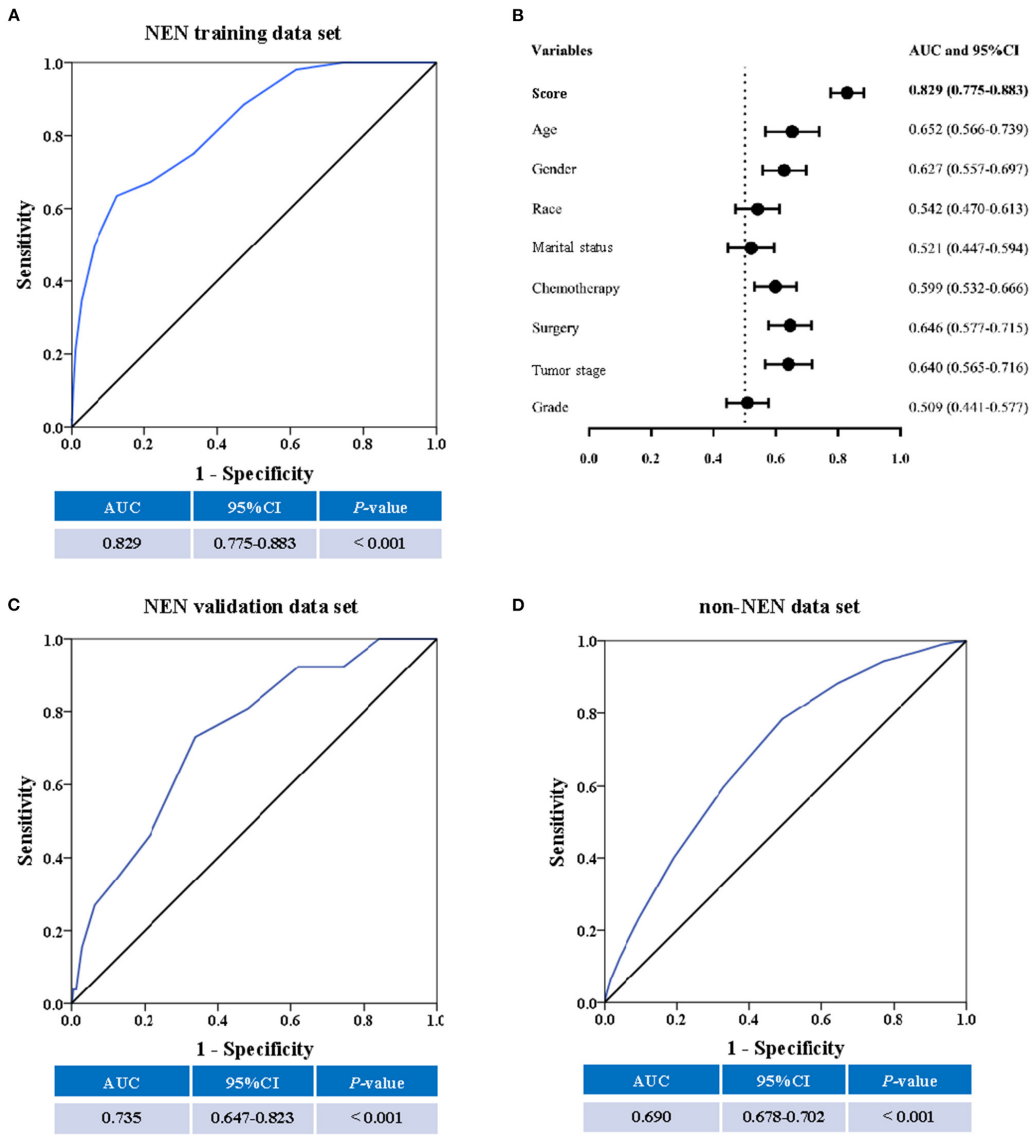
The predictive ability of the score. The receiver operating characteristic (ROC) curve, the area under the curve (AUC) and the confidence interval (CI) suggest a significant discriminatory power of the score when distinguishing between suicide patients from non-suicide patients **(A)**. In addition, the discriminatory power of the score is significantly superior to single factors such as age, gender, race, marital status, chemotherapy, surgical procedure, cancer stage, and cancer grade **(B)**. Moreover, the SEER score could distinguish suicide from non-suicide patients in the validation cohort **(C)**, and the non-NEN cohort **(D)**.

The performance of the score in predicting suicide was evaluated by ROC curve using the training data set. There, we observed that the score could indeed significantly (*P* < 0.001) discriminate suicide patients from non-suicide patients (AUC, 0.829; 95% CI, 0.775–0.883; Figure 4A). To evaluate if the score is significantly superior to each of the single predictors such as age, gender, chemotherapy, surgery, tumor stage, race, material status, and tumor grade, we additionally performed the ROC curves of these predictors (Supplementary Figure 1). These analyses demonstrated that the AUC of the score was significantly higher than that of each single predictor (Figure 4B). In addition, in order to validate the performance of the score, the ROC curve analysis was performed using the validation data set of NEN cohort. We observed that the AUC of the validation data set was 0.735 (95% CI, 0.647–0.823; *P* < 0.001, Figure 4C). Moreover, we tested if this score was also able to predict the suicide of patients in the test data set (non-NEN cohort). The AUC of this test data set was 0.690 (95% CI, 0.678–0.702; *P* < 0.001, Figure 4D). This suggests that this score has acceptable performance and is a stable predictive system for suicide among patients with NEN as well as among patients with other tumor entities. In order to determine the optimal cut-off of this score system, the Youden's index was determined by using the training data set. We observed that the optimal cut-off of the score is 10. This optimal cut-off had a sensitivity of 63.50% and a specificity of and 87.5%.

## Discussion

Suicide is regarded to be a serious public health problem, especially among cancer patients (8, 14). Although, previous studies (7, 8) have determined the incidence of suicide in several cancers within the SEER program, they did not evaluate the incidence of suicide in NEN. In this study, we observed that the PMR of NEN patients is significantly higher than non-NEN patients, especially when the cancer was located in the appendix or pancreas. This suggests that suicide may be more frequent in NEN patients than in non-NEN patients.

We proved, for the first time, that NEN is an independent risk factor for suicide. This is supported by the observation that NEN increases the risk of suicide to 1.8-fold in direct comparison to non-NEN (Table 1). However, the underlying reasons why NEN patients more frequently commit suicide are still unclear. Park et al. (25) suggested that the Bcl-1 polymorphisms of neuron-specific glucocorticoid receptor (NR3C1) may contribute to the susceptibility to suicide in cancer patients. This hypothesis is supported by the concept that dysregulation of NR3C1 leads to depression and suicide by disturbing the function of the hypo-thalamic-pituitary-adrenal axis (26, 27).

Consistent with previous studies (16, 28, 29), we observed that some features of cancer patients such as being young, male, a member of the white race, unmarried, or surgery as well as having cancer in the localized or regional stage increased the risk of committing suicide. However, in contrast to Osazuwa-Peters's study (15), we observed that patients whose tumor was located in lung and bronchus, stomach, or pancreas had a significantly reduced risk of committing suicide, when compared to patients whose tumor was located in colon. In Osazuwa-Peters's study, the authors defined the colon and rectal tumor as an entire cohort. Notably, Tamas et al. (30) suggests that the biological and clinical characters of colon cancer and rectal cancer are different. Hence, we split colorectal cancer into two distinct cohorts, and we observed that tumor located in rectum significantly increased the risk of suicide, when compared to tumor located in colon.

Currently, most cancer patients die of non-cancer causes, such as accidents, heart diseases, and suicide (29). However, the suicide among cancer patients can be directly prevented and several strategies have been established (13, 31, 32). For example, treatments of psychiatric disorders, screening programs for high-risk persons, education for the general public, and restricting access to lethal means (33). Indeed, some studies proved that physician education and restricting access to lethal means, such as guns, domestic gas and barbiturates, could reduce suicide rates (33, 34).

Some screening questionnaires, such as nine-item patient health questionnaire (PHQ-9), hospital anxiety and depression scale (HADS), and Diagnostic and Statistical Manual of Mental Disorders (DSM), have been used for depression screening in cancer patients (13). In addition, the pan-Canadian guideline (35) and the American society of clinical oncology guideline (13) suggest that all patients with cancer should be screened for depressive symptoms at their initial visit and during the treatment until the end-of-life. Notably, most of these tools were originally used to detect depression and anxiety in the general population. They consequently did not include tumor-specific factors, such as tumor stage and grade as well as treatment variables. This may reduce the sensitivity and specificity of these screening tools. Indeed, Hartung et al. (36) demonstrated that the screening performance of both the PHQ-9 and the HADS was limited, when they were applied for depression screening in cancer patients. Thus, our score in combination with current screening tools might successfully identify cancer patients at high-risk of committing suicide.

Our study has two clinical implications: First, prevention of suicide in NEN patients is especially necessary. This conclusion bases on the observation that compared to non-NEN, NEN increases the risk of suicide by 1.8-fold. Thus, a routine screening among NEN patients, especially when the cancer is localized in the appendix or pancreas, may effectively reduce the suicide rate of these patients. In addition, raising awareness of physicians to this topic may be important for patients suffering from pancreatic neuroendocrine tumors (pNET). It is well-known that pancreatic ductal adenocarcinoma (PDAC) is a lethal disease, and the 5-year survival of patients is only around 10% (37). However, the 5-year survival of pNET patients is 50% (3), and there is a high risk that pNET patients actually think they suffer from a PDAC. This may increase the depression and anxiety of these pNET patients. Indeed, the present study suggests that the pancreatic NEN patients have a more than 3-fold higher risk of suicide in comparison with non-NEN patients (Figure 2C). These results imply that patients with longer survival might have an elevated risk to experience depression symptoms and succeed in their suicide attempts when compared to ones with shorter overall survival. Thus, physicians' education may help these patients eliminating depression as well as anxiety, and thus reducing the suicide rate. Second, this study proved that the score (Table 4), which includes general population characteristics (age at diagnosis, gender, race, marital status), tumor characteristics (tumor stage and histologic grade), and treatment parameters (surgery, chemotherapy) can significantly distinguish individuals having committed suicide from those that did not. This supports the hypothesis that incorporation of this score into current screening strategies may prevent suicide among patients suffering from NEN.

## Limitations

Notably, there are some limitations of our studies. First, even though SEER program is an authoritative source of information on cancer incidence and mortality, this database did not identify patients who committed unsuccessful suicide attempts. We, therefore, failed to calculate the incidence of suicide, and only determined the PMR of suicide among NEN cohort and non-NEN cohort. Second, we identified the participants of this study by the cause of death, however, we were unable to evaluate whether there was misclassification bias in our study. Previous studies suggest that suicide may be misclassified as accidents or unintentional death (38, 39). Third, suicide is a complex behavior, and several factors, such as psychiatric disorders, occupation, childhood adversity, family history, and antidepressants can increase the risk of suicide (40, 41). However, the SEER program did not provide these information, and we could not evaluate these factors in this study. In addition, although, this SEER scale has a probability of over 80% to correctly distinguish suicide patients from non-suicide individuals; the false negative rate is 36.5% and the false positive rate is 12.5% when considering 10 as the optimal cut-off point.

## Conclusion

In conclusion, this study, for the first time, demonstrated that patients with NEN have a higher risk of committing suicide than patients with non-NEN. In addition, we developed and validated a suicide score, which can distinguish suicide and non-suicide individuals among these patients. This score may improve the current screening strategies and may reduce the suicide rate especially among patients with NEN.

## Data Availability Statement

The original contributions presented in the study are included in the article/Supplementary Material, further inquiries can be directed to the corresponding author/s.

## Author Contributions

XZ and PG co-supervised the study. LL wrote the manuscript. LL and YS analyzed and interpreted the data. XZ, LL, DZ, ML, and CM performed the critical revision and language check of manuscript. All authors contributed to the article and approved the submitted version.

## Conflict of Interest

The authors declare that the research was conducted in the absence of any commercial or financial relationships that could be construed as a potential conflict of interest.

## Supplementary Material

Supplementary Figure 1 The performance of predictive factors to distinguish between suicide patients from non-suicide patients. The receiver operating characteristic (ROC) curves, the area under the curve (AUC), and the confidence interval (CI) suggest that age **(A)**, gender **(B)**, chemotherapy **(C)**, surgery **(D)**, and stage **(E)** could significantly distinguish suicide patients from non-suicide patients, however, race **(F)**, marital status **(G)**, and grade **(H)** did not have this ability to discriminate.

Supplementary Table 1 Mortality in patients dead from suicide, non-suicide, and all causes.

## Abbreviations

NEN: neuroendocrine neoplasm
SEER: Surveillance, Epidemiology, and End Results
PMR: proportionate mortality ratio
OR: odds ratios
CIs: confidence intervals
β_i_: β coefficient
W_iRef_: weight of the reference category
W_ij_: weight of the other categories
ROC: receiver operating characteristic
AUC: area under the curve
NR3C1: neuron-specific glucocorticoid receptor
PHQ-9: nine-item patient health questionnaire
HADS: hospital anxiety and depression scale
pNET: pancreatic neuroendocrine tumors
PDAC: pancreatic ductal adenocarcinoma.
