# Supplementary figures and images for: Development and Validation of a Score for Screening Suicide of Patients With Neuroendocrine Neoplasms
Source: Front Psychiatry. 2021 Jun 11;12:638152. doi: 10.3389/fpsyt.2021.638152 (PMC8225995; doi:10.3389/fpsyt.2021.638152)

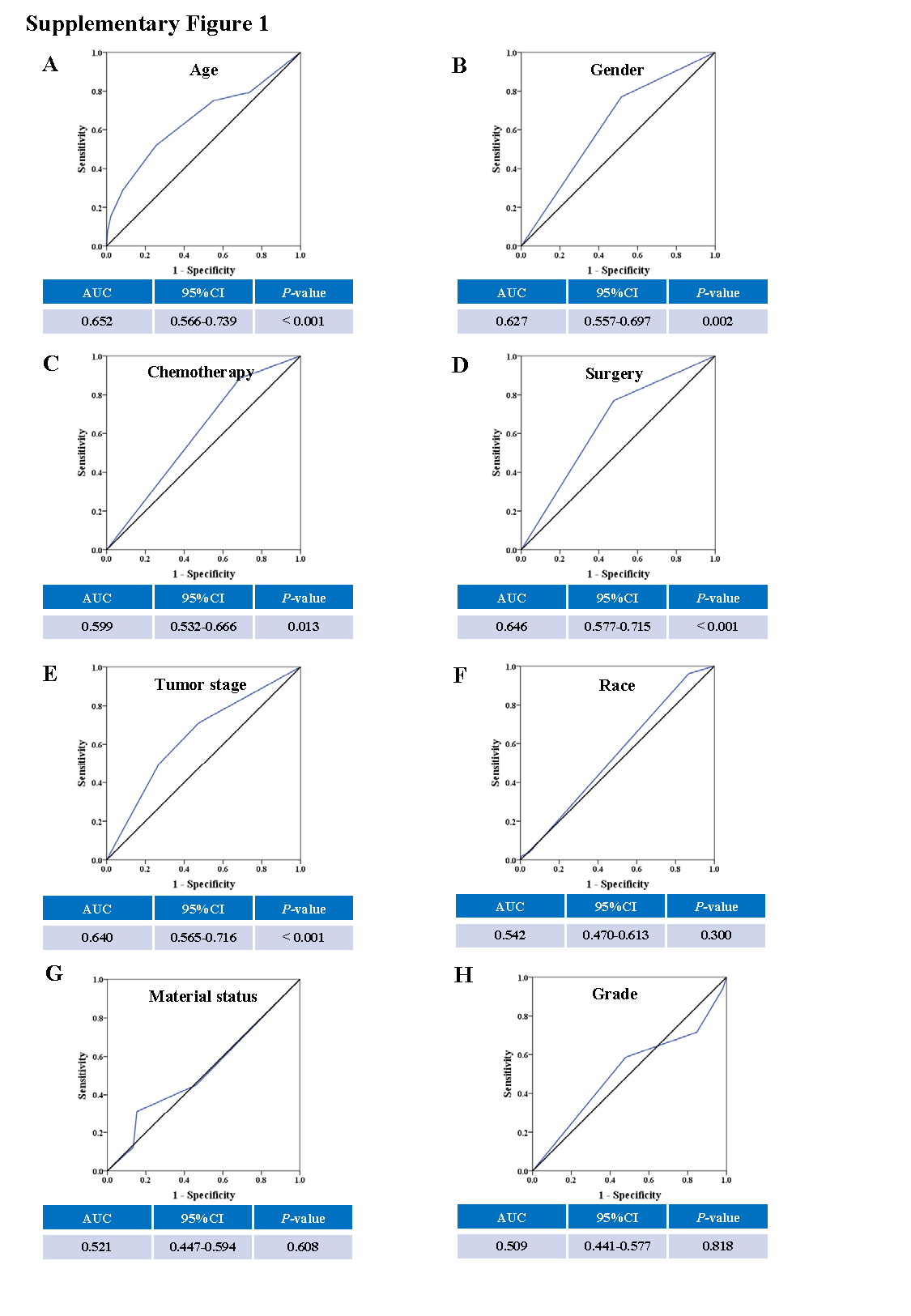

Supplement: Supplementary Figure 1 — The performance of predictive factors to distinguish between suicide patients from non-suicide patients. The receiver operating characteristic (ROC) curves, the area under the curve (AUC), and the confidence interval (CI) suggest that age (A), gender (B), chemotherapy (C), surgery (D), and stage (E) could significantly distinguish suicide patients from non-suicide patients, however, race (F), marital status (G), and grade (H) did not have this ability to discriminate. [file Image_1.TIF]
