# Supplementary material for: Development and Validation of a Score for Screening Suicide of Patients With Neuroendocrine Neoplasms
Source: Front Psychiatry. 2021 Jun 11;12:638152. doi: 10.3389/fpsyt.2021.638152 (PMC8225995; doi:10.3389/fpsyt.2021.638152)
Supplement: Supplementary Table 1 — Mortality in patients dead from suicide, non-suicide, and all causes. [file Table_1.DOCX]

Supplementary Table 1 Mortality in patients dead from suicide, non-suicide and all causes

|  |  | All | | |  | Suicide | | |  | Non-suicide | | |
| --- | --- | --- | --- | --- | --- | --- | --- | --- | --- | --- | --- | --- |
|  |  | Mortality | Lower CI | Upper CI |  | Mortality | Lower CI | Upper CI |  | Mortality | Lower CI | Upper CI |
| NEN | Entire cohort | 1.98347 | 1.95973 | 2.00743 |  | 0.00629 | 0.00503 | 0.00776 |  | 1.97719 | 1.95348 | 2.00111 |
|  | Small intestine | 0.38113 | 0.37074 | 0.39174 |  | 0.00141 | 0.00084 | 0.00220 |  | 0.37973 | 0.36935 | 0.39031 |
|  | Stomach | 0.13736 | 0.13114 | 0.14379 |  | 0.00045 | 0.00016 | 0.00098 |  | 0.13691 | 0.13070 | 0.14333 |
|  | Colon | 0.17018 | 0.16327 | 0.17731 |  | 0.00038 | 0.00014 | 0.00086 |  | 0.16980 | 0.16290 | 0.17692 |
|  | Rectum | 0.15656 | 0.14997 | 0.16336 |  | 0.00098 | 0.00053 | 0.00166 |  | 0.15558 | 0.14900 | 0.16236 |
|  | Pancreas | 0.27244 | 0.26374 | 0.28136 |  | 0.00093 | 0.00049 | 0.00160 |  | 0.27151 | 0.26282 | 0.28041 |
|  | Lung and bronchus | 0.81734 | 0.80209 | 0.83281 |  | 0.00172 | 0.00110 | 0.00256 |  | 0.81563 | 0.80039 | 0.83108 |
|  | Appendix | 0.05734 | 0.05341 | 0.06147 |  | 0.00050 | 0.00020 | 0.00103 |  | 0.05684 | 0.05294 | 0.06096 |
| non-NEN | Entire cohort | 77.61577 | 77.46661 | 77.76515 |  | 0.13089 | 0.12483 | 0.13718 |  | 77.48488 | 77.33584 | 77.63413 |
|  | Small intestine | 0.55274 | 0.54023 | 0.56546 |  | 0.00134 | 0.00079 | 0.00213 |  | 0.55139 | 0.53890 | 0.56410 |
|  | Stomach | 5.12370 | 5.08546 | 5.16215 |  | 0.00782 | 0.00641 | 0.00946 |  | 5.11587 | 5.07767 | 5.15429 |
|  | Colon | 15.68768 | 15.62066 | 15.75493 |  | 0.03194 | 0.02898 | 0.03512 |  | 15.65575 | 15.58879 | 15.72292 |
|  | Rectum | 5.91439 | 5.87338 | 5.95561 |  | 0.01894 | 0.01669 | 0.02142 |  | 5.89544 | 5.85450 | 5.93660 |
|  | Pancreas | 8.17031 | 8.12208 | 8.21874 |  | 0.00826 | 0.00680 | 0.00995 |  | 8.16204 | 8.11384 | 8.21046 |
|  | Lung and bronchus | 41.31957 | 41.21059 | 41.42877 |  | 0.05779 | 0.05376 | 0.06204 |  | 41.26178 | 41.15288 | 41.37090 |
|  | Appendix | 0.23548 | 0.22741 | 0.24377 |  | 0.00059 | 0.00025 | 0.00116 |  | 0.23490 | 0.22684 | 0.24317 |

Rates are per 100,000 and age-adjusted to the 2000 US Std Population; CI: Confidence intervals
